# Anesthesia personnel’s visual attention regarding patient monitoring in simulated non-critical and critical situations, an eye-tracking study

**DOI:** 10.1186/s12871-022-01705-6

**Published:** 2022-05-30

**Authors:** Tadzio R. Roche, Elise J. C. Maas, Sadiq Said, Julia Braun, Carl Machado, Donat R. Spahn, Christoph B. Noethiger, David W. Tscholl

**Affiliations:** 1 Institute of Anesthesiology, University of Zurich and University Hospital Zurich, Raemistrasse 100, 8091 Zurich, Switzerland; 2 Athena Institute, Faculty of Science, VU University of Amsterdam, De Boelelaan 1085, 1091 HV Amsterdam, The Netherlands; 3 Philips Research Eindhoven, High Tech Campus 34, 5656 AE Eindhoven, The Netherlands; 4 Departments of Epidemiology and Biostatistics, Epidemiology, Biostatistics and Prevention Institute, University of Zurich, Hirschengraben 84, 8001 Zurich, Switzerland

**Keywords:** Anesthesia, general, Eye-tracking technology, Patient monitoring, Patient simulation, Situation awareness, Visual attention

## Abstract

**Background:**

Cognitive ergonomics design of patient monitoring may reduce human factor errors in high-stress environments. Eye-tracking is a suitable tool to gain insight into the distribution of visual attention of healthcare professionals with patient monitors, which may facilitate their further development.

**Methods:**

This prospective, exploratory, high-fidelity simulation study compared anesthesia personnel’s visual attention (fixation count and dwell-time) to 15 areas of interest on the patient monitor during non-critical and critical anesthesia situations. Furthermore, we examined the extent to which participants’ experience influenced visual attention and which vital signs displayed on the patient monitor received the most visual attention. We used mixed zero-inflated Poisson regression and mixed linear models to analyze the data.

**Results:**

Analyzing 23 ten-minute scenarios, we found significantly more fixations to the areas of interest on the patient monitor during critical than non-critical situations (rate ratio of 1.45; 95% CI 1.33 to 1.59; *p* < 0.001). However, the dwell-time on the areas of interest did not significantly differ between the non-critical and critical situations (coefficient of − 1.667; 95% CI − 4.549 to 1.229; *p* = 0.27). The professional experience did not significantly influence the visual attention (fixation: rate ratio of 0.88; 95% CI 0.54 to 1.43; *p* = 0.61 and dwell-time: coefficient of 0.889; 95% CI − 1.465 to 3.229; *p* = 0.27).

Over all situations, anesthesia personnel paid the most attention to the vital signs blood pressure (fixation: mean [SD] of 108 [74.83]; dwell-time: mean [SD] of 27 [15.90] seconds), end-expiratory carbon dioxide (fixation: mean [SD] of 59 [47.39]; dwell-time: mean [SD] of 30 [21.51] seconds), and the electrocardiogram (fixation: mean [SD] of 58 [64.70]; dwell-time: mean [SD] of 15 [14.95] seconds).

**Conclusions:**

Critical anesthesia situations increased anesthesia personnel’s visual interaction with the patient monitor. Furthermore, we found that their visual attention focused mainly on a few vital signs. To assist clinicians in critical situations, manufacturers should optimize monitors to convey necessary information as easily and quickly as possible and optimize the visibility of less frequently observed but equally critical vital signs, especially when they are in an abnormal range.

**Supplementary Information:**

The online version contains supplementary material available at 10.1186/s12871-022-01705-6.

## Background

Patient monitoring is an indispensable part of anesthesiologists’ and intensivists’ workplaces [1]. It provides relevant, critical information to the healthcare professionals at the point of care. However, anesthesia and intensive care are areas prone to human errors [2, 3]. Good cognitive-ergonomic design of monitoring devices improves situation awareness and significantly reduces human factor errors in high-stress environments [4, 5].

Exploring how healthcare providers interact with patient monitoring devices is necessary to further improve these technologies. Eye-tracking serves as a powerful tool for objectifying the visual interaction between users and their environment. In recent years, the available eye-tracking devices evolved considerably in terms of size, recording quality, and automated analysis capabilities. Mobile eye-tracking devices have become established tools for gaining insights into cognitive processes such as visual attention, workload, confidence, decision-making and physician awareness [6–10]. Previous studies investigated the healthcare providers’ visual attention regarding patient monitoring systems [8, 11–16]. They found that during real anesthesia cases, healthcare professionals spent about 5% of their time observing the patient monitor [8]. The blood pressure and heart rate seemed to be the most frequently viewed vital signs during simulated anesthesia scenarios [13], and in critical situations, anesthesiologists tend to direct more visual attention toward the patient monitor [14]. However, whether different levels of experience among healthcare providers play a role in the distribution of visual attention on the patient monitor is not yet clear [8, 13, 15, 16].

Using eye-tracking technology, this study investigated the visual attention (i.e., fixation count and dwell-time) on the patient monitor during simulated anesthesia inductions with and without occurring critical incidents. We hypothesized that when managing an emergency situation, anesthesia personnel would pay more visual attention to the patient monitor than when dealing with a non-critical situation. Furthermore, we investigated the influence of the participant’s professional experience, and which vital signs received the most visual attention overall.

## Methods

The Cantonal Ethics Committee of Zurich in Switzerland reviewed the study protocol and issued a declaration of no objection (Business Management System for Ethics Committees Number Req-2020-00059). We obtained written informed consent from all participants to use their data.

### Study design

This investigator-initiated, prospective, single-center, eye-tracking study investigated the visual attention of anesthesia providers regarding patient monitoring during simulated non-critical and critical situations. The eye-tracking data was collected during a high-fidelity simulation study [17]. The simulation study compared an avatar-based visualization technology for patient monitoring with conventional, number- and waveform-based patient monitoring. This eye-tracking study only examined the conventional, number- and waveform-based monitor modality.

According to their availability, we recruited anesthesia teams that consisted of a nurse anesthetist and an anesthesiologist. Each team solved four different ten-minute scenarios. One of the scenarios was mild hypotension after induction without a critical incident (non-critical situation). The three simulated emergencies were severe bronchospasm, malignant hyperthermia, and myocardial ischemia (critical situations). Participants used a randomly determined monitoring modality in each scenario. When the teams performed scenarios with the conventional patient monitor, the randomly determined team leader wore a mobile eye-tracking device (Pupil Invisible by Pupil Labs, GmbH, Berlin, Germany) while managing the scenarios. We used Research Randomizer Version 4.0 (http://www.randomizer.org) to randomize the team leader, the monitoring modality used in the scenario, and the scenario sequence.

### Simulation environment and equipment

We conducted the simulations in a backup operating room that mirrored the study center’s active operating rooms. In addition, we used a state-of-the-art, full human patient simulator (HAL S3201, Gaumard Scientific Company, Inc., Miami, FL, USA) and real medications and airway management tools to increase simulation fidelity. The anesthesia teams used a Philips IntelliVue MX500 (Koninklijke Philips N.V., Netherlands) patient monitor configured to resemble the study center’s regular MX550 and MX800 monitors closely. We tagged the patient monitor in the simulation environment using pre-generated quick response (QR) code, which enables automatic detection by the Pupil Player software (Pupil Labs, GmbH, Berlin, Germany), a prerequisite for automated processing.

### Data collection

Before beginning the scenario, we calibrated the eye-tracking device to the participant. Then, we recorded the participant’s field of view as a video feed using Pupil Invisible (Pupil Labs, GmbH, Berlin, Germany), the mobile eye-tracking device. The device was connected to a cell phone that participants carried in their pocket and served as a power source and storage unit. At the end of each scenario, we uploaded the eye-tracking data to a research server and made backup copies on physical hard drives.

**Fig. 1:**
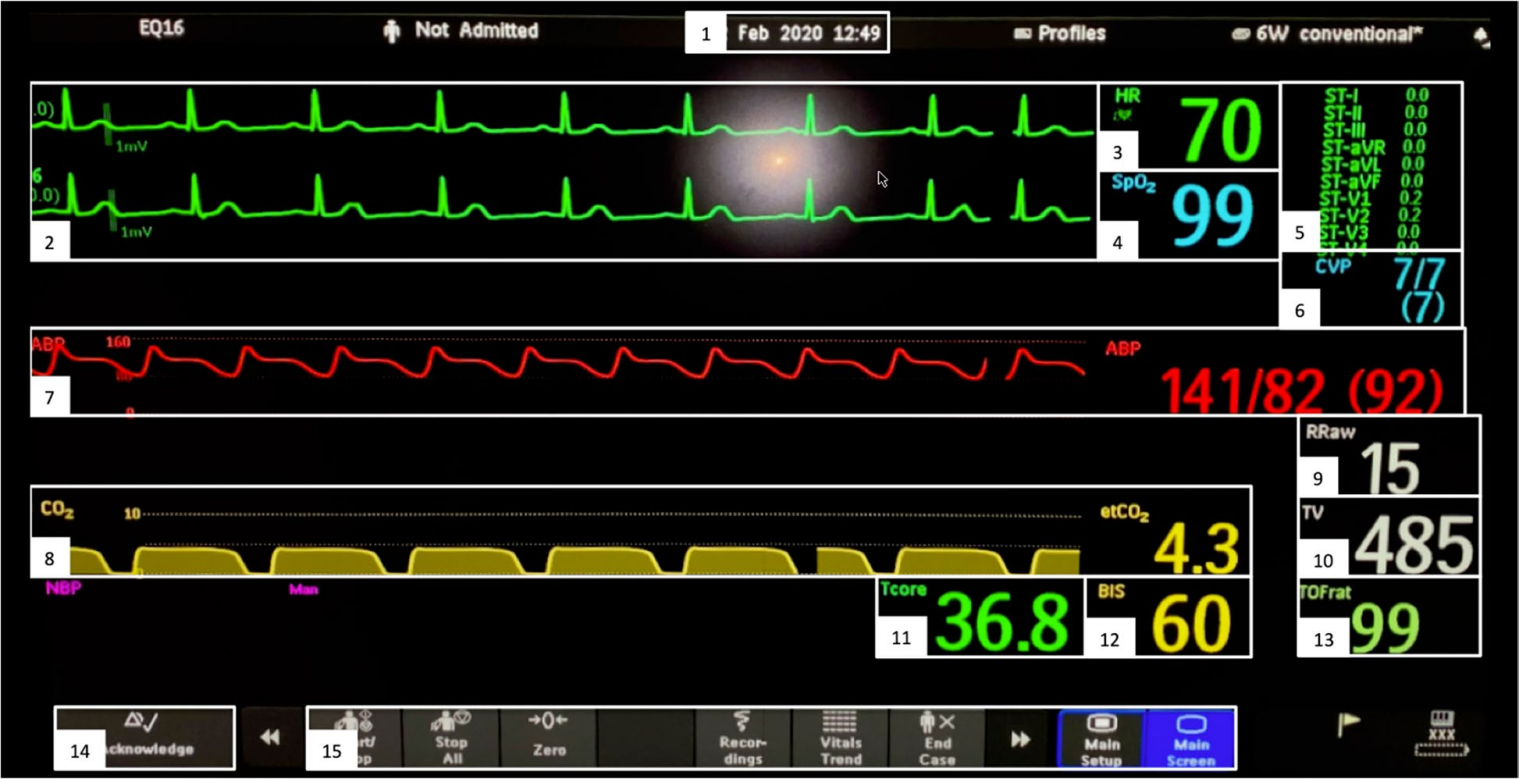
Conventional monitor configuration investigated with eye-tracking in this study. White numbered boxes indicate the predefined areas of interest. 1: Time; 2: electrocardiogram (ECG); 3: heart rate (HR); 4: oxygen saturation (SpO2); 5: ST-analysis of electrocardiogram (ST-Analysis); 6: central venous pressure (CVP); 7: Arterial blood pressure (ABP); 8: end-tidal carbon dioxide concentration (etCO2); 9: respiratory rate (RR); 10: tidal volume (TV); 11: temperature (Temp); 12 bispectral index (BIS); 13: train of four peripheral nerve stimulation (TOF); 14: Button for alarm acknowledgment; 15: Patient monitor settings (PM)

Then, we manually checked the eye-tracking data’s quality and cut the videos to the beginning and end of the 10-minute simulation. We then started the post hoc semi-automated video analysis using Pupil Labs proprietary software Pupil Player (Pupil Labs, GmbH, Berlin, Germany) on an Acer Aspire V15 Nitro laptop (Acer Inc., New Taipei City, Taiwan). Using mentioned software, we traced the patient monitor using the surface Tracker plugin, which automatically detects QR codes in the video sequence. Then we defined 15 areas of interest on the patient monitor screen (Fig. 1). The Fixation Detector plugin enabled us to automatically export all fixations and their durations (dwell-time) on the areas of interest as Microsoft Excel spreadsheets (Microsoft Corporation, Redmond, USA). During the post hoc video analyses, we stopped each recording five times to verify the accuracy of the boundaries of the areas of interest. In case we noticed incorrect boundaries during the recording, we manually corrected the deviation. To validate the accuracy of the Pupil Labs software, we manually determined the distribution between the areas of interest by counting the individual fixations on the areas of interest of about 10% of the eye-tracking data.

### Outcomes

The primary outcome measure was the number of fixations per area of interest. We counted a fixation if participants held their gaze for more than 100 milliseconds on a single location inside an area of interest. We defined the secondary outcome as dwell-time, i.e., the accumulated time spent on an area of interest.

### Statistical analysis

We only included scenarios in which participants used conventional monitoring as this display modality represents the current state of the art method. As the Swiss residency training in anesthesiology requires 5 years of professional practice, we considered trainees as participants with less than 5 years and experts as ones with more than 5 years of clinical experience. We defined the case with mild hypotension after induction as a non-critical situation and the three other simulations (bronchospasm, malignant hyperthermia, and myocardial ischemia) as critical ones.

We calculated the intraclass correlation coefficient (measure for inter-rater reliability for continuous data) to compare the fixation count from the pupil player software and the manual fixation count.

For descriptive statistics, we provide means and standard deviations (SD) as well as medians and interquartile ranges (IQR) for continuous data and numbers and percentages for categorical data. To compare the visual attention (fixations and dwell-times) on the areas of interest in non-critical and critical situations and the visual attention of trainee and expert team leaders, we used mixed models with random intercept per team to incorporate the fact that measurements from the same team are not independent. In detail, for the outcome fixation count, we used mixed zero-inflated Poisson regression models. Regarding the dwell-time, we applied mixed linear models. All models included both the binary experience variable and the binary criticality variable. In the logistic regression part of the mixed zero-inflated Poisson model, we included an area of interest variable. This variable accounts for the fact that not every area of interest (vital sign) had the same importance in each scenario. For example, the electrocardiogram is necessary to diagnose the ST-segment elevations in the myocardial ischemia scenario. In contrast, vital signs that affect ventilation are more important in severe bronchospasm scenario. All statistical analyses were performed using R version 3.6.2 (R Foundation for Statistical Computing, Vienna, Austria). We used Prism 8 (GraphPad Software Inc., California, USA) to create all figures. We considered a *p*-value of less than 0.05 to determine statistical significance.

## Results

**Fig. 2:**
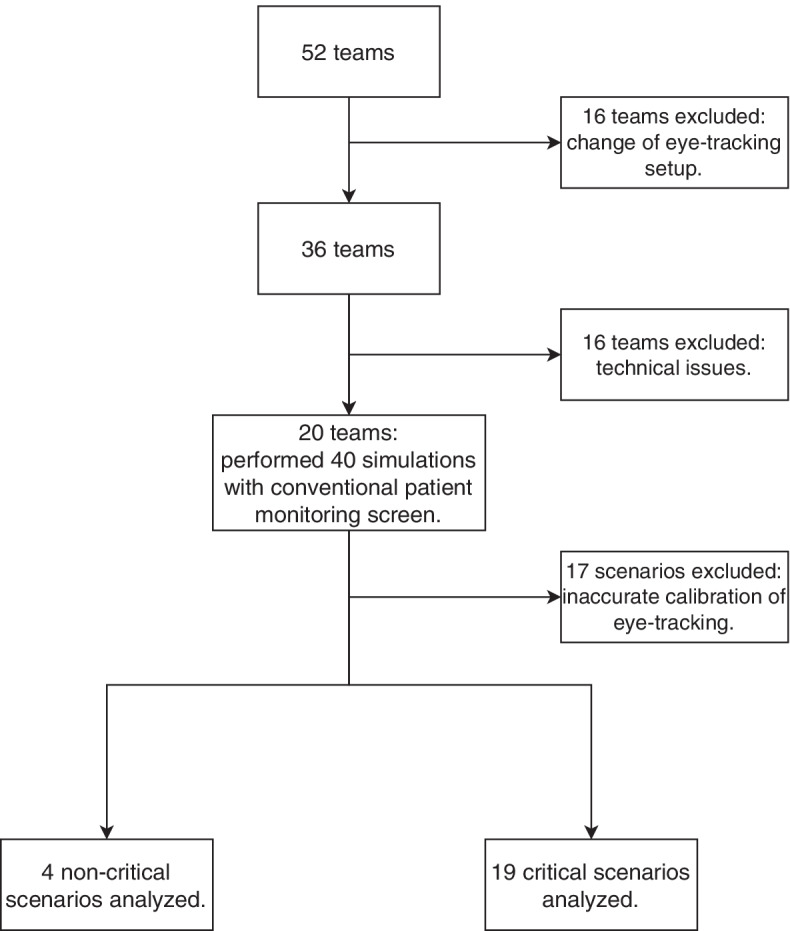
Study setup and exclusion criteria. We changed the QR codes because they were reflective and could not be read by the eye-tracking software. Technical issues were low battery capacity of the eye-tracking device, lack of storage space, an unstable connection between eye tracker and storage device, and problems uploading to the Pupil Player Cloud. The 20 teams that remained after exclusion performed 40 simulations with conventional patient monitoring. After a manual quality check, we excluded 17 scenarios due to eye-tracking calibration inaccuracies (e.g., alternate squinting or prescribed glasses)

This study was conducted in May 2020, including 52 anesthesia teams. Each team performed two scenarios with conventional patient monitoring. We excluded 16 teams as the eye-tracking setup was revised because the laminated QR codes used reflected and were not detected by the eye-tracking software. In addition, we had to exclude another 16 teams because the video material was recorded incompletely due to technical issues. Of the remaining 20 teams that performed 40 scenarios with conventional patient monitoring, we excluded 17 scenarios due to inaccuracies in eye-tracking calibration (e.g., alternate squinting or prescribed glasses). Thus, we analyzed 23 ten-minute simulations performed with conventional patient monitoring. In each simulation, we analyzed 15 areas of interest of the patient monitor (Fig. 1). Figure 2 shows the exclusion criteria in detail. Table 1 displays further study and participants’ characteristics. A comparison of the manual fixation count and the automated fixation count showed an intraclass correlation coefficient across all areas of interest of 0.96 (95% CI 0.70 to 0.99). The absolute numbers and the calculation of the intraclass correlation coefficient are shown in the supplementary material.

**Table 1:** Study and participants characteristics. We considered anesthesia providers with less than five years of professional experience as trainees and anesthesia providers with more than five years of professional experience as experts

Study characteristics	
Simulations conducted with conventional patient monitor and analyzed with eye-tracking, *N*	23
Analyzed non-critical scenarios	4 of 23 (17%)
Analyzed critical scenarios	19 of 23 (83%)
Analyzed areas of interest on the patient monitor	15
Participant’s characteristics	
Team leader, *N*	20
Professional experience in years	mean 6 (min 0; max 33)
Sex, female	11 of 20 (55%)
Job position	
Anesthesia nurse	12 of 20 (60%)
Anesthesiologist	8 of 20 (40%)
Experience level	
Trainee	11 of 20 (55%)
Expert	9 of 20 (45%)

### Overall visual attention on the patient monitor

Within all analyzed simulations, participants had a mean (SD) of 423 (257) fixations per scenario and a mean (SD) dwell-time of 184 (52) seconds on the patient monitor (sum of all fixations/ dwell-times of all 15 areas of interest on the patient monitor). This accounts for 30.7% (184 of 600 seconds) of the simulation time.

### Visual attention to areas of interest in non-critical and critical situations

Regarding the degree of criticality, we found that anesthesia providers had a mean (SD) of 14 (16) fixations with a mean (SD) dwell-time of 11.1 (12.6) seconds per area of interest during non-critical situations compared to a mean (SD) of 24 (38) fixations with a mean (SD) dwell-time of 9.4 (10.1) seconds during critical ones. Comparison of fixations using zero-inflated Poisson models yielded a rate ratio of 1.45 (95% CI 1.33 to 1.59; *p* < 0.001), meaning participants had about 45% more fixations on the areas of interest in the critical than in the non-critical situations. The analysis for dwell-time showed a coefficient of − 1.67 seconds (95% CI − 4.55 to 1.23 seconds, *p* = 0.27). This means that the time spent looking at the areas of interest on the monitor was 1.6 seconds shorter for critical than for non-critical situations. However, this result was not significant.

### Visual attention to areas of interest and professional experience

Regarding the participants’ professional experience, trainees had a mean (SD) of 23 (35) fixations with a mean (SD) dwell time of 9.3 (10.1) seconds per area of interest. Experts had 22 (35) fixations with a dwell-time of 10.3 (11.8) seconds per area of interest. Comparing trainees’ and experts’ fixation count, zero-inflated Poisson models yielded a rate ratio of 0.88 (95% CI 0.54 to 1.43; *p* = 0.61), indicating no evidence for a difference in number of fixations of experts and trainees on the areas of interest. Analyzing the dwell-time, we found a coefficient of 0.89 seconds (95% CI − 1.47 to 3.23 seconds; *p* = 0.27), which means that experts spent a similar time on the areas of interest than trainees.

### Vital signs

**Fig. 3:**
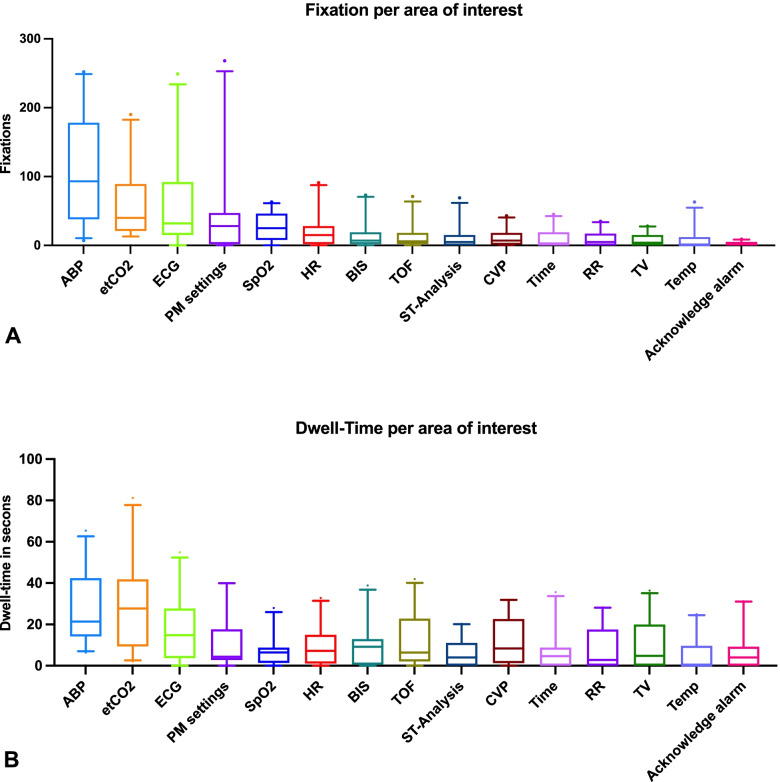
Fixation counts (**A**) and dwell-time (**B**) for all areas of interest in all analyzed scenarios. Box plots are medians with interquartile ranges. Whiskers are 95% confidence intervals. ABP = Arterial blood pressure; etCO2 = end-tidal carbon dioxide concentration; ECG = electrocardiogram; PM settings = Patient monitor settings; SpO2 = oxygen saturation; HR = heart rate; BIS = bispectral index; TOF = train of four peripheral nerve stimulation; ST-Analysis = ST-analysis of electrocardiogram; CVP = central venous pressure; Time = Time display on patient monitor; 12-Lead ECG = 12-lead electrocardiogram; RR = respiratory rate; TV = tidal volume; Temp = temperature; Acknowledge alarm = Button for alarm acknowledgment; Anesthesia providers *n* = 20, Simulations analyzed *n* = 23

Within all simulations analyzed, participants paid the most visual attention to blood pressure (fixation: mean [SD] of 108 [74.83]; dwell-time: mean [SD] of 27 [15.90] seconds), end-expiratory carbon dioxide (fixation: mean [SD] of 59 [47.39]; dwell-time: mean [SD] of 30 [21.51] seconds), and the electrocardiogram (fixation: mean [SD] of 58 [64.70]; dwell-time: mean [SD] of 15 [14.95] seconds). Figure 3 displays the fixation count and dwell-time of all analyzed areas of interest as boxplots.

## Discussion

By using eye-tracking technology we found that anesthesia providers observed the patient monitor for about 31% of the simulation time, with an increase in monitor fixations of approximately 45% during critical situations. However, dwell-time did not differ significantly when comparing critical and non-critical situations. Further, the participants’ different professional experiences did not significantly affect the distribution of visual attention. The vital signs, blood pressure, end-expiratory carbon dioxide, and electrocardiogram received the most visual attention.

During critical anesthesia events, the staff actively increased their number of fixations on the patient monitor to gain sufficient awareness of the situation. However, despite more fixations during such events, we did not find a significant increase in dwell-time. This seems somewhat counterintuitive but may be explained by a different use of the patient monitor in the care of stable and unstable patients. We believe that in volatile critical events, rapid vital sign changes require more but shorter fixations on the patient monitor to maintain good situation awareness and manage the events successfully. In the case of a hemodynamically unstable patient, the anesthesiologist must recognize the problem within seconds, understand the meaning or severity of the problem, realize where the situation will go, develop a therapy concept, and implement this concept immediately. Then, the anesthesiologist must check the applied therapy for success and continuously reevaluate the patient to maintain situation awareness [18, 19]. These stressful situations with tremendous time pressure may lead to more frequent but shorter fixations of the corresponding vital signs on the patient monitor and may explain the findings of this study. To further explore this assumption, sequence analyses that examine both scan patterns and performance of manual tasks, for example, drug administration, should be performed in future studies.

In our analyses, the professional experience level did not affect the fixation count and dwell-time on the patient monitor. Literature provides conflicting results on this topic. Schulz et al. postulated a tendency for more experienced anesthesiologists to devote less time to the patient monitor during critical incidents [14]. Also, Tourangeau et al. found differences in visual perception of the situation between trainees and experienced anesthesiologists [20]. Consistent with our results, in a recent study by Grundgeiger et al., the professional experience level did not affect the monitoring-related visual attention in simulated and real anesthesia inductions [8]. Nevertheless, there may be different reasons why anesthesia personnel check the patient monitor during critical incidents. Inexperienced anesthesia providers may randomly check the vital signs on the monitor in a saccadic way to get an idea of the situation at hand (data-collection-driven visual perception). Experienced personnel may correctly perceive the situation more quickly (knowledge/experience-driven perception) but check the relevant vital signs more frequently or longer to anticipate the future course or the consequences of their actions. Evidence for this assumption is provided by a study in which inexperienced anesthesiologists performed more saccades (defined as rapid movement between fixations) between AOIs during a “cannot intubate/cannot oxygenate” simulation. In contrast, experienced anesthesiologists spent more time on the relevant parameters with fewer saccades between the AOIs [20]. Furthermore, Desverges et al. demonstrated that experienced anesthesiologists were more likely to monitor blood loss than inexperienced ones in a simulated postpartum hemorrhage [13]. After all, those findings suggest a difference in visual attention depending on the level of experience. Perhaps this study’s small sample size may have masked the linkage between visual perception and expertise, or the measurements used (fixation count and dwell-time) are not sensitive enough. A sequence analysis, in which scan patterns and manual tasks are analyzed together, may provide better information about the use of the patient monitor. Further studies are needed to investigate this hypothesis.

During our simulations, anesthesia providers looked at the patient monitor approximately 31% of the time. This proportion is similar to other simulation studies [14, 21] but drastically different from observed visual attention in the real operating room [16]. Studies reported dwell-times on the patient monitor during real surgery to be approximately 5% [16]. One might assume that this difference is because the simulation studies did not include the intraoperative anesthesia maintenance phase, which is less dynamic than the anesthesia inductions or critical situations. However, a study examining simulated and real inductions showed that the dwell-time on the patient monitor of anesthesiologists was between 3 to 5% [8], which puts the above argument into perspective.

Analyzing the areas of interest on the patient monitor, we found that the vital sign blood pressure received the most visual attention in both non-critical and critical situations. This is consistent with a recent study [13]. However, we expected the respiratory vital signs such as oxygen saturation, tidal volume, and respiratory rate to receive more visual attention. This may be explained by the additional sonification used in monitoring the oxygen saturation. The modulating pitch of the heart rate sound reliably indicates oxygen saturation [22]. This showed that perception and situation awareness depend not only on visual data but also on various data sources that we can perceive with our senses, such as hearing. Further intelligent sonification of vital signs could be helpful and is already investigated [23, 24].

This study has several limitations. First, we had to exclude more than half of the teams from the analyses due to technical issues or poor data quality. Although we used one of the latest mobile eye-tracking devices available on the market, we faced several challenges in recording the data: Calibration of the eye-tracker for ocular pathologies (e.g., alternating squinting or wearing prescription glasses), battery life issues, device slipping off participants’ faces during physical tasks (e.g., manual resuscitation), fogging of glasses and cameras while wearing a face mask (an essential requirement during the COVID-19 pandemic). This shows that despite the massive development of eye-tracking hardware and software in recent years, the technology is still error-prone, and it is difficult to obtain high-quality video recordings, which are essential for reliable and semi-automated analyses. Second, we chose small, closely spaced areas of interest. To obtain reliable eye-tracking data, the areas of interest should not be too close together to compensate for eye-tracker inaccuracies [25, 26]. However, our manual count of fixations showed good agreement with the automatically counted fixations. We believe the plan surface of the patient monitor is a main reason for the good accuracy despite the small and close areas of interest. A different study using Pupil Labs ((Pupil Labs, GmbH, Berlin, Germany) hard- and software achieved excellent accuracy with the patient monitor but relatively poor accuracy with non-planar areas of interest [27].

Third, we conducted this study in a tertiary care hospital in central Europe. Results may vary under different conditions and in other parts of the world. Fourth, all simulations inherently entail a bias [28]. They may not reflect the actual critical incident’s psychological and temporal pressures and dynamics. Apparently, this influences visual attention distribution as well [8, 9]. Interestingly, a recent study has reproduced results from a randomized control trial in a simulation study [29]. Hence, this may question the importance of simulation bias. However, this study possesses several strengths. First, we reduced selection bias by balancing participant selection and randomizing the scenario sequence. We sought to address the inherent limitation of authenticity that resonates with all simulation-based studies [28] through our in situ study design and efforts to represent clinical reality as closely as possible. Further, we used eye-tracking hardware that was no more intrusive than wearing regular glasses to produce high-quality video footage. Finally, we replaced error-prone human analysis with semi-automated analysis to help ensure high-quality data.

## Conclusion

Critical situations increased anesthesia professionals’ visual attention to the patient monitor. The anesthesia personnel mainly visually focused on blood pressure, end-expiratory carbon dioxide value, and electrocardiogram. We advise patient monitor manufacturers to best match the medical professionals’ needs to optimize information transfer. Furthermore, we believe that the visibility of less frequently observed but equally critical vital signs should be improved, especially when they are in an abnormal range.

## Supplementary Information

**Additional file 1:** **Table 1.** Manual fixation count, Pupil Player software fixation count and intraclass correlation.

## Data Availability

The datasets used and/or analyzed during the current study are available from the corresponding author on reasonable request.

## Abbreviations

AOI: Area of interest
IQR: Interquartile range
SD: Standard deviation
