# Supplementary material for: Anesthesia personnel’s visual attention regarding patient monitoring in simulated non-critical and critical situations, an eye-tracking study
Source: BMC Anesthesiol. 2022 May 30;22:167. doi: 10.1186/s12871-022-01705-6 (PMC9149329; doi:10.1186/s12871-022-01705-6)
Supplement: Supplementary file 1 — Additional file 1: Table 1. Manual fixation count, Pupil Player software fixation count and intraclass correlation. [file 12871_2022_1705_MOESM1_ESM.docx]

### **Supplementary material**

Table 1 Manual fixation count, Pupil Player software fixation count and intraclass correlation. Values are numbers.

| Video 1 | Pupil_Soft | Rater1 | Video 2 | Pupil_Soft | Rater1 |
| --- | --- | --- | --- | --- | --- |
| ABP | 122 | 109 | ABP | 119 | 97 |
| Acknowledge alarm | 4 | 3 | Acknowledge alarm | 5 | 7 |
| BIS | 23 | 17 | BIS | 19 | 11 |
| etCO2 | 94 | 71 | etCO2 | 123 | 109 |
| CVP | 18 | 12 | CVP | 15 | 4 |
| ST-Analysis | 11 | 10 | ST-Analysis | 11 | 14 |
| ECG | 37 | 31 | ECG | 56 | 43 |
| HR | 15 | 9 | HR | 49 | 42 |
| PM settings | 56 | 37 | PM settings | 35 | 24 |
| RR | 14 | 9 | RR | 9 | 4 |
| SpO2 | 16 | 11 | SpO2 | 55 | 39 |
| Temp | 0 | 5 | Temp | 10 | 13 |
| TOF | 20 | 11 | TOF | 0 | 2 |
| Time | 30 | 18 | Time | 6 | 9 |
| TV | 26 | 17 | TV | 11 | 6 |
| Intraclass correlation coefficient | 0.95 (95% confidence interval 0.87 to 0.98 | | Intraclass correlation coefficient | 0.96 (95% confidence interval 0.79 to 0.99) | |
| Overall Intraclass correlation coefficient | 0.96 (95% confidence interval 0.70 to 0.99) | | | | |
